# Vibrotactile -Feedback Device for Postural Balance Among Malocclusion Patients

**DOI:** 10.1109/JTEHM.2020.2990527

**Published:** 2020-04-27

**Authors:** Bhornsawan Thanathornwong, Wattana Jalayondeja

**Affiliations:** 1 Faculty of DentistrySrinakharinwirot University 37692 Bangkok10110Thailand; 2 Faculty of Physical TherapyMahidol University 26685 Nakhon Pathom73170Thailand

**Keywords:** Force plate, occlusion, body posture, center of pressure

## Abstract

Multiple studies have suggested that some associations exist between occlusal factors and postural alterations. Objectives: This study aimed to evaluate the effectiveness of a vibrotactile posture trainer device, comprised a wearable device containing an accelerometer sensor to measure the angle of the neck flexion (input) and provided real-time vibrotactile biofeedback (output) for postural balance among patients with malocclusion. Methods: Twenty-four subjects were divided in 3 groups based on occlusion and using Angle’s classification. Each group consisted of 8 patients for class I, II and III malocclusion. The Posture Trainer System was used for feedback concerning neck flexion angles when higher than 15 degrees. A 4-week training program to adjust posture balance in 2 axes (flexion-extension, lateral-flexion) was applied in activities for daily living. The assessments in this study were comprised of neck flexion angles from the Posture Trainer System and the center of pressure ($\text{N}\cdot \text{m}$) using a force plate. The effects of a vibrotactile posture trainer (baseline vs. post-training test) were evaluated using the paired t-test and were assumed to be significant at p < 0.05 (two-side). All analyses were conducted using the Statistical Package for Social Sciences, Version 21.0 (SPSS, Chicago, IL, USA). Results: Neck flexion angles and center of pressure significantly decreased post-training by the Posture Trainer System among patients with class II malocclusion. No changes in the above parameters post-training were found in class I and class III. Conclusion: The results demonstrated that patients with class II malocclusion training by the Posture Trainer System lowered neck flexion angles and COP compared with pre-training. Clinical Impact: Feedback by the Posture Trainer System can help improve the postural balance in class II malocclusion.

## Graphical abstract

This study aimed to evaluate the effectiveness of a vibrotactile posture trainer device, comprised of a wearable device containing an accelerometer sensor to measure the angle of the neck flexion (input) and provide real-time vibrotactile biofeedback (output), for postural balance among patients with malocclusion. Results of a study in 24 subjects showed that that patients with class II malocclusion training by the Posture Trainer System experienced lowered neck flexion angles and COP compared with pre-training.

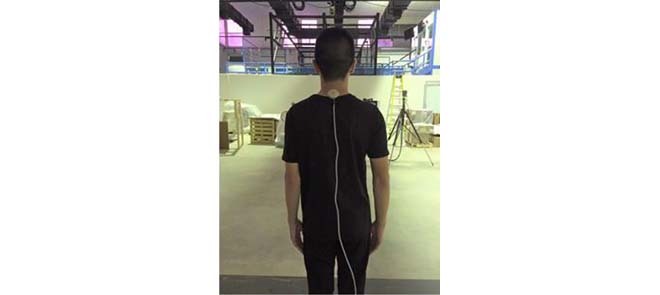

## I. Introduction

Currently, oral and maxillofacial surgery treatments are increasingly linked to dental treatments, especially by surgeons and orthodontists. The goal of treatment is to fix dentofacial anomalies or undesirable facial structures to create a more natural look and correct occlusion. Dental occlusion refers to the alignment of teeth and the way that the upper and lower teeth fit together. Malocclusion is most often hereditary. It causes tooth overcrowding or abnormal bite patterns. There are different categories of malocclusion: Class I malocclusion is the most common. The bite is normal, but the upper teeth slightly overlap the lower teeth. Class II malocclusion, known as retrognathism or overbite, occurs when the upper jaw and teeth severely overlap the bottom jaw and teeth. Class III malocclusion, called prognathism or underbite, occurs when the lower jaw protrudes or juts forward, causing the lower jaw and teeth to overlap the upper jaw and teeth.

Surgical treatments are aimed at correcting anomalies of facial and occlusions so that any changes are clearly visible. The results of the post-treatment often showed that some patients can retain satisfactory conditions for a long time. However, for some patients, the symptoms included less obstructive interferences, less masticatory efficiency, muscular and occlusion balance and less centric occlusion to centric relation discrepancy reappear. In this surgical treatment, only the facial structure was fixed, while other body parts, as well as the factors causing dentofacial abnormalities, were negligent [1]. Many studies investigated the body structures that affect occlusion, focused on abnormal human body structures, acquired both genetically and nongenetically. The findings indicated that, in general, people with unusual body structures are also associated with abnormal facial structures and occlusion due to the fact that both structures are connected with muscles that help to counteract the imbalance [2]. The upright position of the head is maintained by a balanced tension between the craniocervical bones, myofascial structures and dental occlusion. Finally, the upper cervical spine acts as the mediator between head and trunk and forms an anatomically and functionally interrelated system.

The studies on the relationship between the biomechanics of the dental occlusion have continued to grow in number in the literature [4]. Indeed, researchers generally analyze the effects of dental occlusion on the body structure. The results reported a significant correlation between distal jaw position, sagittal mandibular length, and increased cervical lordosis [3]. The cited studies, using a balance platform, showed that subjects with Class II occlusion exhibited an anteriorly displaced posture, whereas subjects with Class III occlusion exhibited a posteriorly displaced posture. Regarding the cervical vertebrae, nearly one half of patients with a Class I or II, showed a marked cervical lordosis whereas Class III exhibited abnormal kyphosis [1]. Moreover, a study by Arumugam *et al.* [5] indicated that patients with severe malocclusions most commonly had a forward head and neck posture. This forward head and neck posture also significantly correlated with Class II skeletal pattern. This was supported by Alwarawreh *et al.* [6], who tested the inclination of body symmetry and found that the body tilt of patients with severe malocclusion, such as severe protrusion or severe distal jaw positions, was highly affected.

Posture is commonly assessed using a grid or plumb line and with the patient in a static standing position; however, within the clinical research, this becomes less accuracy in terms of postural sway. Several studies related to the hypothesis of the effect of occlusion on the postural balance and used a force plate to analyze the relationship. The center of pressure (COP) was used as an indicator of the body posture measurement, a universally accepted method. It can be easily understood and is the key value used to assess the straight position [7], [8]. The COP is the point where the total sum of a pressure field acts on a body, causing a force to act through that point. The total force vector acting at the COP is the value of the integrated vectorial pressure field [9]. An accelerometer sensor is used to assess neck flexion angles. From recent studies in Thailand, researchers developed Posture Trainer Systems (patent no 7659, 8548 and 8549) and collected operating data to assess neck flexion angles at work. The key component is a microcontroller that works with an accelerometer and vibrators. This device vibrates when it senses neck parts of users tilting beyond proper position or for a long period of time during work. Results indicated that people who used the device had a lower average of muscle activity in the upper trapezius muscle than those who did not [10].

This study aimed to investigate the effects of the Posture Trainer System on the neck flexion angles and COP from the force plate in various classes of malocclusion. The research intended to answer whether this device could be of help in balancing the body posture and whether or not it could be used in conjunction with dental occlusion to preserve satisfactory treatment conditions for a longer time. Furthermore, the results acquired from this study could be used as primary data for treatment planning among patients with dentofacial anomalies, which may require balance adjustment of the body posture to help sustain the most effective treatment.

## II. Methods

### A. Participants and Design

In this pilot intervention study, a repeated measures design with a four-week intervention program was used. Twenty-four patients (mean age 23.4 years, ranging from 20 to 30 years; 20 female, 4 males) with 3 classes of malocclusion were divided in 3 groups (8 patients for each group): groups 1, 2, 3 for patients with molar relationship class I, II and III occlusion, respectively. Inclusion criteria included a diagnosis of molar relationship occlusion by an orthodontist. There was no evidence of congenital disease involving the musculoskeletal system, no deformities in the body structure from accidents, and no history of orthodontic treatment was noted. Patients were excluded if they had scoliosis.

**FIGURE 1.**
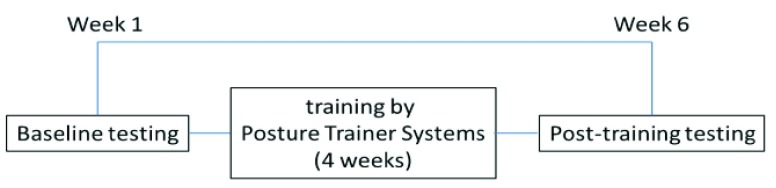
Schematic of the study procedure.

The assessments were performed at baseline (within one week before the beginning of the intervention), post-training (within one week after the last training session) (Figure 1). All patients also received a Posture Trainer System in their home to improve posture, static and dynamic balance and activities of daily living for 6 hours daily (6 hours per day, five days per week, for 4 weeks). The outcomes of measurements were determined in static posture. Subjects needed to stand for 20 seconds. The primary outcome measures were the neck flexion angle and secondary outcome were mean values of COP. The study was approved by the institutional Ethics Review Board and written consent forms were provided by all participants.

### B. Posture Trainer System

**FIGURE 2.**
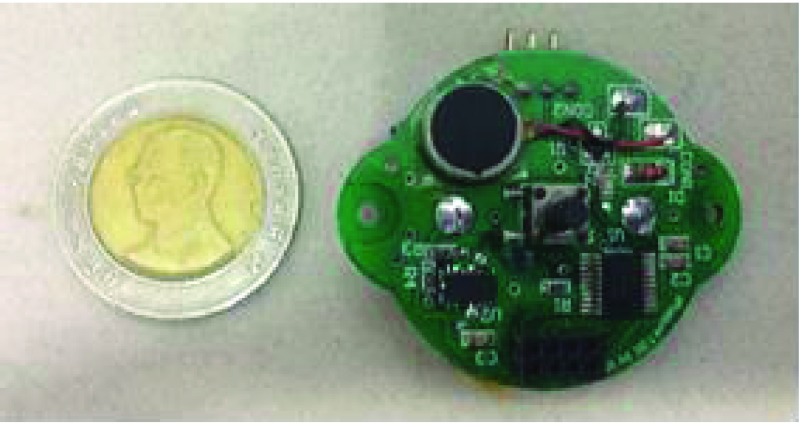
Posture trainer system devices.

The feedback system for training contained ADXL345 3-axis accelerometer sensors with high resolution (13-bit) measurement at up to ±16g and a 12.5-400Hz bandwidth response. This sensor could track neck flexion angles on 2 axes and provided Xout and Yout, in the digital IO voltage range of 1.8-2.5V. A data acquisition card (13-bit resolution) was used to amplify and convert voltage to digital signals. The vibrotactile feedback was initiated when the neck flexion angles was higher than 15 degrees (Figure 2). The goal of the sensors was to develop a home-based monitoring and intervention system that would provide vibrotactile feedback for training. Neck flexion angle defines the angle between global vertical and the vector pointing from C7 to occiput-cervical joint 1. The small-sized and light weight device was attached to the neck using sticky tape at the C7 vertebra level. This location is appropriate to measure the neck angle. On the ergonomics view point, over 15 degrees of neck flexion is claimed to be awkward posture. This angle is usually used in ergonomic measurement (tool) like RULA (Rapid upper limb assessment) to determine risk of musculoskeletal disorders [11]. This sensor could be used standalone when the user required only feedback or was connected to a computer when required to record neck flexion angles.

### C. Training Protocol

The training program followed these objectives: (1) to improve body posture and static balance and (2) to improve dynamic balance. The system provided feedback when the neck flexion angles were higher than 15 degrees.

### D. Assessments

**FIGURE 3.**
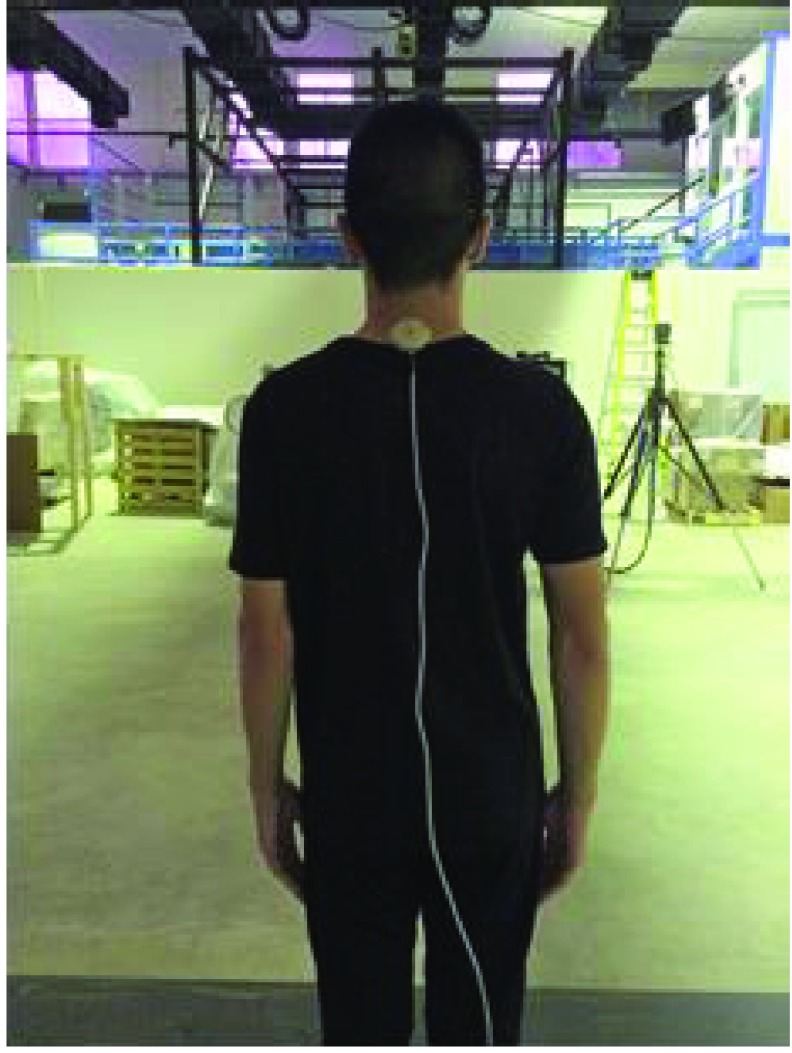
Mounted posture trainer system.

Assessments included tests of balance and neck flexion angles. Balance tests were obtained from a force plate (BERTEC Corporation, model FP9090-15-TM-2000, USA). The posture trainer sensor was attached to the subject’s upper back at the spinous process of the 7th cervical vertebra (Figure 3). The subjects were asked to stand on the force plate with bare feet using the standard posture (International Society of Posturography, ISP): heels close together and toes 30 degrees apart. They were asked to stand normally and look straight ahead. The Bertec forceplate was imbedded in the floor. Therefore, there was no height effect of the forceplate on the balance of the subjects. The degree obtained from the Posture Trainer System (degrees) was recorded as well as the COP values taken from the force plate ($\text{N}\cdot \text{m}$) simultaneously for 20 seconds.

### E. Data Analysis

The data was analyzed using a paired t-test between the baseline and post-training tests and significance was set at p < 0.05 (two-sided). All analyses were conducted using the Statistical Package for the Social Sciences, Version 21.0 (SPSS, Chicago, IL, USA).

## III. Results

All participants completed the study using the Posture Trainer System and all of the evaluations were reported. The demographics of the participants are summarized in Table 1. No adverse events were reported using these sensors in the homes of the participants. All patients subjectively reported that the sensors were easy to understand and convenient, the device was light weight, and was uncomplicated to use.

**TABLE 1:** Patients’ Characteristics (n=24)

	Mean	SD	Range
Age [yr]	23.4	4.3	20–30
Height [cm]	161	5.6	153.0–167.0
Weight [kg]	50.85	10.1	48.0–70.0
BMI [kg/m^2^]	22.1	4.5	19.7–29.9

Post-training, patients with Class II malocclusion received feedback from the system with a significantly lower neck flexion angles and center of pressure (Table 2, 3) (p < 0.05). There was no significant difference between the neck flexion angles and COP found post-training in both Class I and II malocclusion.

**TABLE 2:** Neck Flexion Angles (Degrees) Baseline Test and Post-Training Test With the Posture Trainer System in 3 Classes of Patients With Malocclusion

Occlusion	Neck flexion angles from Posture Trainer System (degrees)		p
	Baseline test	Post-training test	
Class I (n=8)	5.63±4.57	5.63±3.66	1.000
Class II (n=8)	8.37±3.96	5.00±3.70	.007^*^
Class III (n=8)	21.38±18.02	6.13±1.46	.053

^*^ Test results were statistically significant at a level of 0.05

**TABLE 3:** Center of Pressure ($\text{N}\cdot$m) Baseline Test and Post-Training Test With the Posture Trainer System in 3 Classes of Patients With Malocclusion

Occlusion	Center of Pressure from Force Plate (N$\cdot$m)		p
	Baseline test	Post-training test	
Class I (n=8)	25.34±10.22	16.18±6.30	.061
Class II (n=8)	22.29±5.81	13.83±4.41	.003^*^
Class III (n=8)	20.76±11.09	14.74±3.04	.129

^*^ Test results were statistically significant at a level of 0.05

## IV. Discussion

In terms of upright human bodies, the spinal column forms an ‘S’ shaped curve; with the skull bent 90 degrees in the center so that front half stayed level with the ground and the back half on top of the spinal column. The stability of posture was maintained by the muscles, which exerted traction all around the body and the periphery of head. On the sides, the mastoid process is attached to the shoulders and the neck muscles. The shoulders are also attached to pelvis, which was reshaped with concave front surface. Behind the attachment of muscles are thick occipital prominences all over the spine. In front, the muscles are attached to the chest and the clavicle, pulling the hyoid bone down to the mandible, also attached to the zygoma and the temporal bone. Thus, all the muscles of body are attached to bones to balance the head over the spinal column, which work in unison. Any disturbance in one component may result in changes to other components. The mandible is attached anteriorly to skull through teeth and posteriorly through temporomandibular joint. Any change in position of mandible is compensated for in occlusion through the teeth or at the temporomandibular joint. Centric relation controls the posture of the mandible and all eccentric movements begin at this position. Excessively retruded centric relation position can cause forward head posture, an adaption to protect the airway. When the head is centered on top of spinal column it is balanced, when it is shifted forward bending forces are produced all along the length of the spine. The inner portion of shoulders shifts forward and rotate inwards, while the outer portions stick out. The realignment of all the structures includes the pelvis rotating down and in front to thrust the abdomen forward under the head and the chest sinks back down. Every centimeter of forward head posture triples muscle tensions which function as myofacial chains but put strain on the intervertebral joints. Firstly, the muscles of posture are affected and then the muscles farthest from source are affected. Forward head posture pulls the mandible towards the maxilla, decreasing freeway space, shortening suprahyoid muscles, and stretching the infrahyoid muscles and creating tension in muscles of mastication and leading to malocclusion. It is extremely important to evaluate and treat posture simultaneously while treating occlusion [12]. Many studies refer to a supposed correlation between malfunction of the masticatory apparatus and an anterior positioning of the head [13]–[16]. These correlations should make clinicians consider the advisability of integrating the evaluation and treatment of postural defects at the same time they are correcting discrepancies in the masticatory system [13], [17]–[19].

Nowadays, in changing the subject’s habits to improve their body posture, sensors play an important role. In one related study, the researcher developed a device consisting of a sensor measuring the degree of movement of a participant’s neck while the software provided real-time feedback to the participant about incorrect posture [14]. The best part of this device was the computer program, providing feedback in real-time and enabling users to make body posture adjustment and assisting users to perform appropriate body movements leading to muscle memory.

In this study, the Posture Trainer System was used to analyze the degree of neck movements, with feedback data provided by vibration when the neck flexion angles of the participants went beyond a defined range. In this study, the limit was set at 15 degrees on 2 axes: flexion and lateral flexion. The results suggested when training was received that a molar relationship class II malocclusion, the body pattern at an angle of neck flexion related to body balance with statistical significance, i.e., when the body was balanced, the COP would be lower as well.

Worldwide, the prevalence of Angle Class II malocclusion in mixed and permanent dentitions was 19.56% and 23% respectively [20]. The study results are valuable for the development of a method for efficient treatment of class II malocclusion using the Posture Trainer System. The results of this study suggest that posture training may be a beneficial therapy for most patients who are interested in improving their posture. There was also a program contract that included training at home with the Posture Trainer System 6-hour supervised practice sessions for 4 weeks while participating in the study. Patients who hold their head in a neutral position relative to the shoulders have a high probability of experiencing improvement in their balance and posture [19]. The Posture Trainer System responded using vibration. In this study, after using a sensor attached at the 7th cervical vertebrae of the participants indicated that the average degree of movement decreased when the feedback compared with the average degree of the baseline test, especially at the angle of neck flexion. The decreased change in COP indicated less body swing, which increased the capability of the body balance [21]. This was consistent with the results, indicating that the body was balanced when training was received, which differed from the statistical significance.

In addition, the findings also indicated that in class II malocclusion, the changing degree of the body tilt and changing values of the COP ($\text{N}\cdot \text{m}$) were low and suggesting a normal and balanced posture. Many studies do assert that the occlusion exerts an influence on pressures applied through the feet [22], [23]. Moreover, this balanced body posture could be found in a balanced occlusion. It also confirmed the results from studies of occlusion affecting body posture, i.e., findings on a well-balanced mandible, which suggested the smooth contraction of the throat muscles and the sternocleidomastoid muscle on both sides and helped, reduce body swing [24]. Also, changes or modifications of the parts that affect any other body part connected with muscles ensure a reaction by the nervous system as well, influencing the COP and gait stability as exhibited through different body postures [23], [25].

Gadotti *et al.* [26], analyzed the head posture and electromyographic (EMG) activity of the anterior portion of temporal and masseter muscles bilaterally among subjects with different dental occlusion as classified according to Angle. The results indicated that the EMG responses of temporal and masseter muscles tended to be modified in Angle Class II subjects who presented more frequently the occurrence of forward head posture. Also, Nobili *et al.* [27] studied this correlation by means of posturography on a group of 50 patients belonging to every Angle’s malocclusion. In this, which was designed as a case series and lacked a matched control group, the subjects were asked to stand on the balance platform and to perform five different tests. The authors concluded that subjects with Class II malocclusion exhibited a forward body position, whereas body position in subjects with Class III malocclusion was posteriorly displaced. A possible causal explanation for this association was given by Solow and Sandham [28] who used the term “soft-tissue stretching” differences in craniofacial morphology could be explained by the stretching of the soft tissue layer of the skin when the head is bent backward.

Children with skeletal class II showed a significantly higher extension of the head upon the spinal column compared to children with skeletal class I and skeletal class III. Recently a correlation was also found between cervical lordosis and mandibular divergency [29]. A study by Lippold *et al.* [30] among children 3.5 to 6.8 years old found a relationship between class II molar relationship occlusion and body posture. The children in this group had a poorer body posture than other children. Therefore, observing occlusion during childhood is recommended to prevent future disorders of the body structure. The body tilts for class I, II and III molar relationship occlusion indicated no correlation between body posture and COP. This was consistent with findings by Alwarawreh *et al.* [6], who found that only severe occlusion was correlated with the symmetry of the body at an angle of inclination with any statistical significance, while less severe occlusion had no effect on the symmetry of the body. Therefore, the suggestion to treat malocclusions, especially among adolescents, in order to correct the head posture has a high probability of experiencing improvement.

Feedback devices are widely used today. Many types of feedback systems are available, e.g., vibration, audio and visual from computer screens, etc. Alakahone *et al.* [31], for example, designed a program to measure the degree of body movement with vibration as a real-time feedback system for use in their study. Two targeted groups were tested: the visible and invisible groups. The participants in these 2 groups were fitted with a sensor on their back and tested both with and without feedback data. The results indicated that the visible group exhibited less body movement both with and without feedback data. Quite possibly, the sensor on their back might provide inaccurate or incorrect information.

The usage of vibration-type feedback data to improve postural control was found to be successful in many studies. Gopalai and Senanayake [32] collected vibration-type of feedback data by attaching a sensor to the backs of the participants while standing on a wobble board. The group with feedback data had greater improvement in postural control than the group without. Similar results were also found in a study by Alahakone and Senanayake [33]. A vibrotactile feedback prototype could significantly reduce sway in both eyes-closed and eyes-open conditions when subjects were standing in the tandem Romberg position. Wong *et al.* [34] used a feedback device with an audio warning to control patients who presented adolescent idiopathic scoliosis. They found that audio-type feedback data succeeded in helping the patients to control their body posture as well as those of hard orthoses. Its main advantage over hard orthoses; however, was data recording, which showed the cooperation of patients when wearing the device. The patients were also satisfied with it because the audio-type feedback device was smaller than the available hard orthoses, which helped to reduce effects on social interaction. It also was not due to spinal muscular atrophy, rib bone deformity, peeling skin or gastrointestinal disorders. Dozza *et al.* [35] conducted an experiment using a feedback device with an audio warning. Their study suggested that the group receiving feedback data showed a statistically significant reduction in forward/backward and left-side/right-side movements and demonstrated a better balanced posture.

Wu [36] studied a real-time visual feedback device on a computer screen to determine the center of gravity of the body and used this in balance posture training for elderly patients presenting peripheral neuropathy. The balance posture assessment indicated that the use of such a real-time feedback devices could provide better balance posture training results and helped patients gain more confidence in balancing effectively. Earlier successful uses of real-time feedback devices in patients with hemiplegia and vestibular disorders were also reported. Research by Milosevic [37], was conducted through experimentation using audio-visual feedback to help control appropriate body posture when participants moved from a set balanced position, indicating that feedback data when received by the experimental group significantly helped them to improve their posture.

However, the research findings have revealed that the average changes in degrees of body posture and COP of all 3 classes of molar relationship occlusion did not differ significantly and were slightly related. This was consistent with a study by Perinetti *et al.* [9], [38] stating that class I, II and III molar relationship occlusion were somewhat associated with the balance posture. Therefore, concluding that any tendency could occur for the balanced posture in this group of occlusions was impossible. The researcher proposed that further research should not only set the criteria to categorize the severity of occlusion but to also add a factor concerning the balanced posture during the data collection stage.

## V. Conclusion

The results demonstrated that patients with class II malocclusion training by the Posture Trainer System lowered neck flexion angles and COP compared with pre-training.
